# Risk factors for relapse in non-infectious cryoglobulinemic vasculitis, including type I cryoglobulinemia: a systematic review

**DOI:** 10.3389/fimmu.2023.1215345

**Published:** 2023-07-07

**Authors:** Nithya Rajendran, Puteri Maisarah Rameli, Hanaa Awad

**Affiliations:** ^1^ Department of Immunology, Beaumont Hospital, Royal College of Surgeons in Ireland (RCSI) Hospital Group, Dublin, Ireland; ^2^ Department of Acute Medical Assessment Unit (AMAU)/ General Internal Medicine (GIM), St. James’s Hospital, Trinity College Dublin, Dublin, Ireland

**Keywords:** small-vessel vasculitis, cryoglobulinemia vasculitis, cryoglobulinemia, non infectious cryoglobulinemia vasculitis, non-HCV related cryoglobulinemia, essential cryoglobulinemia, systematic review, relapse

## Abstract

**Background:**

Data on non-infectious cryoglobulinemic vasculitis (NICV) is scarce, especially concerning the management of relapses, which are troublesome. We aimed to investigate risk factors for relapse in NICV.

**Methods:**

A systematic literature search of CINAHL, Embase, MEDLINE, Scopus, and the Web of Science databases was implemented until April 2023. Eligible studies included randomized control trials, observational studies, and case series with ≥4 patients. Two reviewers independently extracted data and assessed the quality of the eligible studies.

**Results:**

A total of 3,724 articles were retrieved from a database search, with 27 studies meeting the inclusion criteria for review. Most studies (n = 23) detailed relapses, with the time to relapse varying between 1 and 80 months. The relapse rate was reported at 28% in Type I NICV and ranged from 22% to 60% in mixed NICV. Risk factors for relapse in NICV were identified based on the cryoglobulin subtype and correlated with clinical and immunological responses to varying treatment regimens. Type I NICV with an associated lymphoproliferative disorder exhibited a response-relapse pattern. Cutaneous and articular involvement and incomplete clinical and immunological responses to treatment, particularly corticosteroid monotherapy and occasionally rituximab, influence the risk of relapse in Type II and Type III NICV.

**Conclusion:**

Our findings underscore the significance of attaining both clinical and immunological responses and identifying risk factors for relapse in NICV. Appropriate risk stratification for NICV patients is essential for the successful implementation of effective treatment strategies.

**Systematic review registration:**

https://www.crd.york.ac.uk/prospero/, identifier CRD42023408140.

## Background

Cryoglobulins are immunoglobulins (Ig) that undergo precipitation at temperatures below 37°C and re-dissolve upon rewarming (1). Cryoglobulinemia is a heterogeneous condition defined by the presence of circulating cryoglobulins in the sera and their deposition into small to medium blood vessels, causing chronic inflammation and end-organ damage (2). According to Brouet et al., cryoglobulinemia can be classified into three subtypes: Type I cryoglobulins comprising isolated monoclonal Ig, usually IgM, and this can be associated with plasma cell dyscrasias; Type II cryoglobulins comprising polyclonal IgG and monoclonal IgM with rheumatoid factor (RF) activity; and Type III cryoglobulins comprising polyclonal IgG and polyclonal IgM with RF activity, commonly referred to as mixed cryoglobulinemia (MC) (2, 3). Cryocrit, defined as the relative volume of the precipitate as a percentage of the total serum volume, is elevated in Type 1 and lowest in Type III cryoglobulins (4). Rocatello et al. recently introduced a novel concept of idiopathic hypocryoglobulinemia in patients with clinically suspicious vasculitis who have negative cryoglobulins by standard techniques. Through a modified precipitation technique in a hypoionic medium, a subset of patients are found to have trace levels of circulating polyclonal Type III cryoglobulins (hypocryoglobulins), redefining the diagnostic pathways for cryoglobulinemia (5).

Cryoglobulinemic vasculitis (CV) is an immune complex-mediated small- to medium-vessel vasculitis. The prevalence of CV is rare, estimated at approximately 1 per 100,000 worldwide. It is commonly observed in patients between 45 and 65 years of age, with a predominance of a female-to-male ratio of 3:1 (3). CV can be classified into infectious CV (ICV), where Hepatitis C virus (HCV) infection accounts for 90% of cases, and non-infectious CV (NICV), which is associated with autoimmune diseases and hematological malignancies, mainly B-cell lymphoproliferative disorders (6).

A triad of clinical presentations, including fatigue, arthralgia, and palpable purpura, are typically found in type II and type III cryoglobulinemia. In comparison, type I cryoglobulinemia is usually associated with conditions of the skin such as Raynaud’s phenomenon, ulcers, and gangrene (3). The literature describing the clinical characteristics and effective treatment of NICV is limited. The pathogenesis of NICV is less understood compared to ICV, but it is well acknowledged that chronic inflammation resulting from autoimmune conditions stimulates B-cell proliferation through complement and macrophage activation, subsequently inducing cryoglobulinemia (2, 3).

Various treatment approaches have been used in CV, which involve the use of corticosteroids (CS), rituximab (RTX), azathioprine (AZA), and plasmapheresis (PLEX) either individually or in combination. However, the treatment response in CV is variable, and treatment failures have been reported. In type I cryoglobulinemia, targeted therapies addressing the underlying lymphoproliferative disorder did not affect relapses (7). In HCV-related mixed cryoglobulinemia (MC), successful eradication is achieved using direct-acting antivirals (8). The combined administration of pegylated interferon α or ribavirin and/or novel antiviral drugs has also been effective in inducing remission in most cases of MC (9). However, despite successful treatment, there are instances of treatment failures, which can result in persistent disease manifestations or relapses (8). These treatment failures emphasize the importance of understanding the underlying disease mechanisms in different subtypes of cryoglobulinemia and developing more effective therapeutic strategies.

Clinical relapse is defined as the reappearance or recurrence of vasculitic clinical manifestations following the achievement of a complete treatment response. A complete immunological response refers to the normalization of the C4 complement level and the reduction/disappearance of cryoglobulins in sera. Only one study examined possible predictors for early relapse, where the absence of achieving a complete immunological response influenced relapse (10). Pulmonary involvement, gastrointestinal involvement, renal insufficiency, and advancing age >65 years were notable factors independently associated with death (11). To date, there is limited data on risk factors for relapse in NICV. Therefore, this systematic review aims to explore possible risk factors for relapse to guide effective patient management.

## Materials and methods

This systematic review was conducted according to ‘Preferred Reporting Items for Systematic Reviews and Meta-Analyses (PRISMA) (12). We registered our protocol with the International prospective register of systematic reviews (PROSPERO) on 14 March 2023 (PROSPERO registration CRD42023408140).

### Search strategy

A literature search was implemented between March and April 2023 across key electronic databases Embase and MEDLINE by a trained librarian and one reviewer through OVID access (https://ovidsp.ovid.com) for thoroughness—MEDLINE (EBSCO Interface), MEDLINE (Ovid Interface), CINAHL (Complete Interface), CINAHL (EBSCOHost Interface), Embase (Ovid Interface), Web of Science, and Scopus using agreed key search terms. Articles published from the inception of databases (Embase 1974; Medline 1946) until 2023 were included. Only articles published in English were reviewed. Key search terms used in the literature search were: cryoglobulinemic vasculitis, cryoglobulinemia vasculitis, essential mixed cryoglobulinemia, non-infectious cryoglobulinemia vasculitis, and relapse.

### Study selection

#### Eligibility criteria/inclusion criteria

1. Articles on clinical features, investigations, treatment, and outcomes, including relapse, prognosis, and mortality in patients with NICV
2. Studies including randomized control trials (RCT), observational studies, and case series with ≥4 patients

#### Exclusion criteria

1. Articles not published in English
2. Duplicates
3. Letters to the editor, opinions, commentaries, communication, reviews and meta-analyes, conference abstracts, and case series with a sample size of fewer than four patients

Two reviewers (NR and PR) thoroughly examined the electronic databases for relevant titles and abstracts. Articles suitable for further review were independently explored for full text or abstract. Eligible studies matching inclusion criteria were analyzed, and references within eligible studies were assessed retrospectively as potential additions to our review. Any disagreement on article selection was achieved through consensus-building face-to-face meetings with a third reviewer.

### Data extraction

Two independent reviewers (NR and PR) performed an in-depth analysis of eligible articles meeting inclusion criteria using a standardized data extraction form [**S1**]. The data extraction form included: 1) article title, author information, publication date, and country of publication; 2) study design; 3) sample size and demographics; 4) clinical characteristics, investigations, and management; 5) outcome of interest—clinical manifestations for relapse, risk factors of relapse, and treatment adverse event(s).

### Quality assessment

The quality of included studies was assessed independently by two reviewers (NR and PR) using the Newcastle-Ottawa Scale (NOS) for cohort studies (13) (**S2**) and Version 2 of the Cochrane risk-of-bias tool for randomized trials (RoB 2) for RCTs (14) (**S3**). A STROBE checklist was used to assess bias risk in cohort studies (15) (**S4**).

## Results

**Figure 1:**
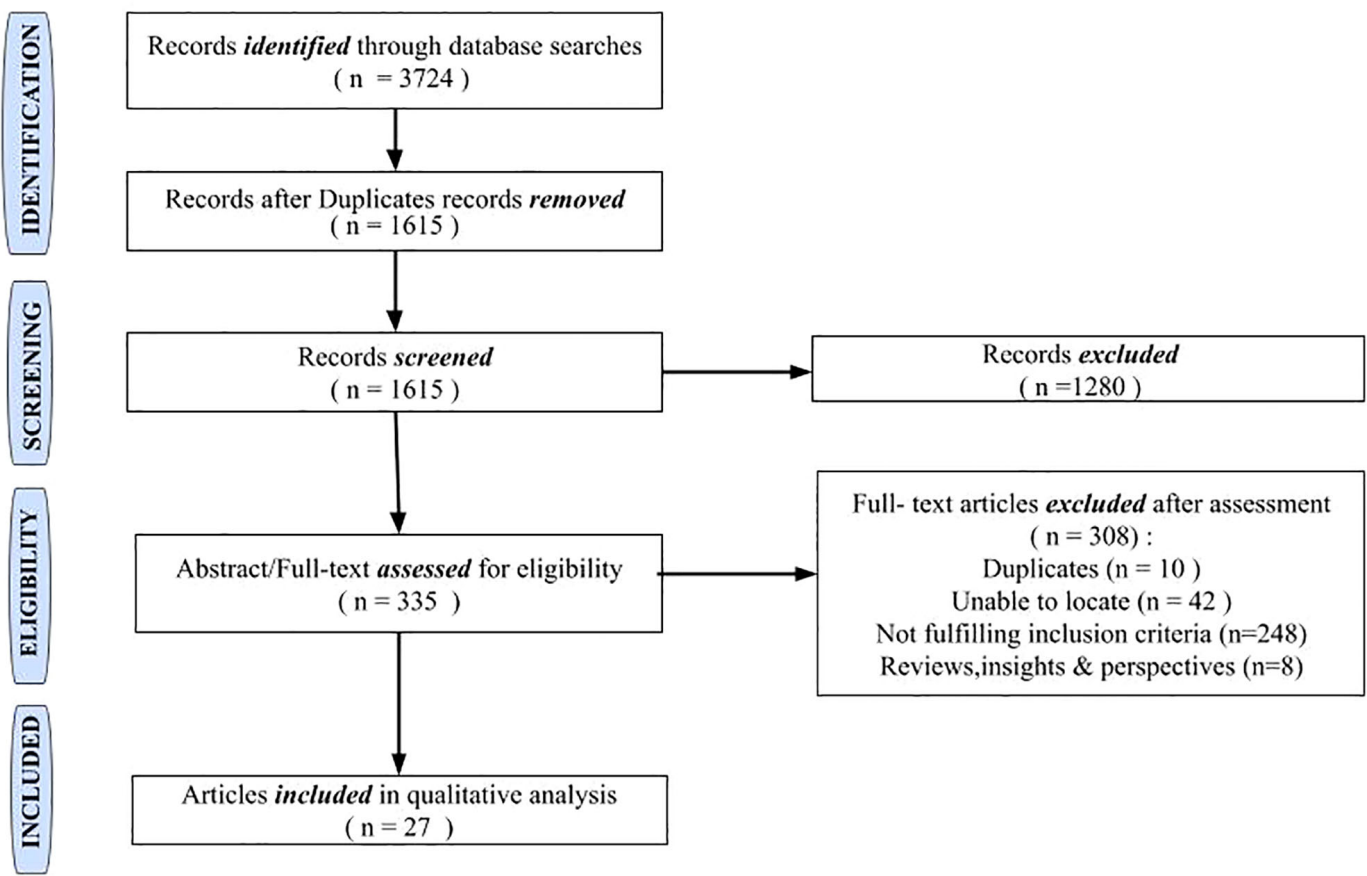
Summary of the study selection process for systematic review.

A total of 3,724 records were retrieved from the database search (**Figure 1**). Following deduplication, 1,615 records were screened through a review of titles. A total of 335 records were retained for full-text assessment following the exclusion of 1,280 records that did not fulfill our inclusion criteria. A total of 27 studies comprising randomized controlled trials (n = 1), non-randomized clinical trials (n = 1), observational studies (n = 23), and case series ≥4 (n = 2) were included in the final review. Due to the scarcity of clinical trials and large prospective cohort studies on NICV, we were unable to perform a meta-analysis.

For this review, the sample size has been adjusted with the NICV cohort segregated from the total population in 10 studies (16–25). The total population from the included studies was 1,519, with a mean of 60.76; the study population ranged between 1 and 266. This review encompassed several studies that included a broad spectrum of patient characteristics in the context of relapses in CV, shedding light on its clinical characteristics, therapeutic approaches, and prognosis. The patient population included age groups ranging from 21 to 91 years, with a female gender preponderance. Studies included a range of countries: France (fourteen studies), Italy (six studies), the USA (two studies), and one study each from Brazil, Greece, and Poland. Multiple countries were involved in two studies (**Table 1**).

**Table 1:** Characteristics of studies included in the review.

Author	Year	Study Design	Country	Population (n)	Age Mean ± SD/ Median (Range)	Gender (n%)	Risk factors
Zaja	2003	Clinical trial	Italy	15;3 EMC*	66 ± 2	66.6% Female	All three EMC cases experienced relapses
Bryce	2006	Retrospective	USA	8; 6 NICV with LPD*	65 ± 11.5	62.5% Male	MALT Lymphoma with Sjögrens syndrome
Cruz	2006	Case Series	Brazil	9	54.44 ±17.01	77.7% Female	EMC cases experienced more relapses than other etiological NICV associations
Saadoun	2006	Retrospective	France	133; 116 NICV*	59.5 ± 16.6	65% Female	NICV refractory cases—Efficacy of RTX + Belimumab reported; Purpura on relapse
Matignon	2009	Retrospective	France	20; 9 pSS, 1 BCL, 10 EMC	59.8 ± 12.12	75% Female	Renal ± extra-renal relapses observed. CV relapse with purpura, concomitant BCL
Terrier	2010	Retrospective	France	23	66 ± 13	74% Female	pSS more likely to experience relapse
Foessel	2011	Retrospective	France	33; 13 EMC, 14 CTD 4 HM	EMC 62.8 ± 12.2 Secondary 57.3 ± 13	53.8% Male 75% Female	CS Monotherapy; RTX flare
De Vita	2012	RCT	Italy	57; 4 NICV*	63.27 ± 10.78	80.7% Female	Patients randomised to the non-RTX group failing treatment switched to RTX (RTX-switch group)
Terrier	2012, 2013	Retrospective	France	242	62.6 ± 14.5	69% Female	Non-RTX + CS regimen
Terrier	2013	Retrospective	France	64	65.4 ± 11.4	56% Female	Post RTX CV flare 13%
Terrier	2014	Retrospective	France	145	61.2 ± 11.8	73% Female	Purpura; Cutaneous necrosis; Articular involvement Incomplete clinical and immunological response after treatment; CS monotherapy
Néel	2014	Retrospective	France	227; 36 with features of MC	63 (41–85)	55.6% Male	With underlying LPD, demonstrated response-relapse pattern necessitating multiple treatment modalities
Michaud	2015	Retrospective	France	29	57 ± 16.4	69% Female	Not reported
Retamozo	2016	Prospective	Spain/ Italy	21*	50.9 ± 13.9	90.4% Female	Not reported
Zaidan	2016	Retrospective	France	230; 80 MCGN+	62.6 ±14.1	62.5% Female	Renal ± extra-renal relapses were reported. GFR <60 ml/min; non RTX + CS regimen
Colantuono	2017	Retrospective	Italy	37; 2 EMC*	72(35 -82)	78% Female	The 2 EMC cases relapsed with low-dose RTX
Galli	2017	Prospective	Italy	175; 160*	66 (55–74)	78% Female	Not reported
Sidana	2017	Retrospective	USA	102; 98*	59 (31–91)	54% Male	Cryocrit level reported predicts symptom burden
Lobbes	2018	Case Series	France	4	73 ± 9.9	50% Female	Non-IgM Type I CV when treated with CS monotherapy.
Marson	2018	Retrospective	Italy	159; 42 NICV*	68 (IQR 62–74 )	73.6% Female	Not reported
Argyropoulou	2020	Retrospective	Greece	71*	50 (21–75)	97% Female	Reported factors influencing the risk of Lymphoma
Boleto	2020	Longitudinal study	France	742; 266 NICV*	55 (IQR 38–68)	37.2% Male	Not reported
Desbois	2020	Retrospective	France	50; 1 EMC*	83	100% Male	RTX-associated flare where patients shared characteristics of type II IgM k with RF activity; renal involvement; BCL; Increased CG level and low C4 complement level
Lesniak	2021	Retrospective	Poland	3	62.3 ± 4.04	100% Female	Refractory cases with underlying EMC
Fenoglio	2022	Prospective	Italy	11#; 8 MC	63.8 (54–79)	63% Male	Type II CV with renal involvement—Proteinuria
Pouchelon	2022	Retrospective	France/Italy/Germany/Spain	26 **^**	56.5 (46.5–65.0)	58% Female	Refractory to RTX -Type II Mixed CV Monoclonal IgMκ with incomplete immunological response
Roubertou	2022	Retrospective	France	213; 142 SLE CG+ 21 CV+	29.2 ± 12.8	87% Female	Not reported

*NICV cohort isolated from the total; ^ NICV cohort from the original study; # Inclusive of type I; BCL, B-cell lymphoma; CG, cryoglobulins; CS, corticosteroids; CV, cryoglobulinemic vasculitis; EMC, essential mixed cryoglobulinemia; GFR, glomerular filtration rate; LPD, lymphoproliferative disorders; IQR, interquartile range; MCGN, mesangio capillary glomerulonephritis; MC, mixed cryoglobulinemia; NICV, non-infectious cryoglobulinemia vasculitis; pSS, primary Sjögren's syndrome; RTX, rituximab; SLE, systemic lupus erythematosus.

The most frequently reported non-infectious etiological associations were connective tissue diseases accounting for 41.8% (n = 636), predominantly primary Sjögren’s Syndrome (pSS) and systemic lupus erythematosus (SLE), and haematological diseases comprising 27.6% (n = 416) encompassing MGUS and haematological malignancies (HM). Approximately 26.1% consisted of EMC (n = 397), whose underlying etiology was unidentified.

The included studies consistently found prevalent cutaneous manifestations in the patient population at baseline, while articular involvement, neuropathy, and nephropathy were observed to varying degrees. Immunological findings such as elevated cryoglobulin levels, low complement C4 levels, and positive RF activity in MC were commonly detected. Treatment strategies varied and included CS, RTX, cyclophosphamide (CYC), AZA, PLEX, and others. An in-depth summary of the study and patient characteristics are outlined in **Tables 2**, **3**.

**Table 2:** Summary of study characteristics on risk factors for relapse in type 1 cryoglobulinemic vasculitis.

Type 1 Cryoglobulinemic Vasculitis								
Study	Aetiology	Clinical manifestations	Immunological features	Intervention with follow-up duration (months)	Clinical response	Immunological response	Treatment-associated adverse events	Outcome
Terrier (2013) (n = 64)	MGUS 44% HM 56%	Cutaneous 86% Articular 28% Neuropathy 44% Nephropathy 30%	Median CG 1.55 g/L Median C3 0.89 Median C4 0.09	MGUS-related CV: CS ± PLEX/AKA/Chloraminophene/CYC/RTX/AZA HM-related CV: CS ± RTX based/AKA/Bortezomib/±Thalidomide Follow-up 46.2 months	MGUS 35%, HM 19% in remission	NR in NHL (LPL)	13% of severe infections 13% RTX worsening vasculitis	MGUS 39% relapse, 26% refractory to first-line therapy HM 48% relapse, 21% refractory to first-line therapy 1 RTX-associated CV flare; Death 7%
Néel (2014) (n = 36)	NMMG 36% HM 64% (WM 52%, MM 17.39%, CLL 4.34%, NHL 23%)	Cutaneous 75% Articular 19% Neuropathy 47% Nephropathy 31% (MPGN 75%)	CG IgM 69.44% Mean CG 2.5 g/L IgG 30.55%. κ 72%	HM—cytoreductive regimen NMMG—non-cytoreductive regimen ± PLEX Follow-up 63 months	Not assessable for CV - heterogeneous therapeutics for underlying LPD	Not described	Treatment AE and infections 27%	Older age; Renal involvement frequent with IgG CG; Death 25%
Sidana (2017) (n = 102)	LPD 92% HCV 3.9% Unknown 3.9%	Cutaneous 63% Articular 24% Neuropathy 32% Nephropathy 14%	CG IgG 53% IgM 39% Polyclonal IgG/ IgM 5% Median cryocrit HM 10%; MGUS 5%	CS/AKA/RTX alone or combination; novel myeloma drugs ± PLEX/ASCT Follow-up 50 months	CR 66% PR 14% NR 21%	PR in HM and MGUS (Cryocrit 8% and 1%)	Not described	Neurological involvement impacts survival outcome
Lobbes (2018) (n = 4)	MGUS 25% MGUS + pSS 25% MM 25% BCL 25%	Cutaneous100% Articular 75% Neuropathy 25%	CG 726–6,762 mg/L CG IgG-κ 14.7; IgG-λ (5.6 & 22.6); IgA-λ 13 g/L	Bortezomib ± low dose CS	Noted in 75%	CR in cases 1,3 PR in cases 2,3	Pneumonia Worsening neuropathic pain	Relapse 100% between 4 and 18 months; Death 25% with uncontrolled CV, 50% due to underlying disease

AE, adverse event; AKA, alkylating agents; ASCT, autologous stem cell transplant; AZA, azathioprine; BCL, B-cell lymphoma; CR, complete response; CS, corticosteroids; CG, cryoglobulins; CLL, chronic lymphocytic leukaemia; CV, cryoglobulinemia vasculitis; CYC, cyclophosphamide; HCV, hepatitis C virus; HM, hematological malignancy; Ig, immunoglobulins; LPD, lymphoproliferative disorders; LPL, lymphoplasmacytic lymphoma; MGUS, monoclonal gammopathy of undetermined significance; MM, multiple myeloma; MPGN, membranoproliferative glomerulonephritis; NHL, non-Hodgkin’s lymphoma; NMMG, non-malignant monoclonal gammopathy; NR, no responders; PLEX, plasmapheresis; PR, partial response; RTX, rituximab; pSS, primary Sjögrens syndrome; PR, partial response; RTX, rituximab; WM, Waldenström macroglobulinemia.

**Table 3:** Summary of study characteristics on risk factors for relapse in type 2 & 3 mixed cryoglobulinemia.

Type 2 & 3 Mixed cryoglobulinemia								
Study *only NICV sample	Aetiology	Clinical manifestations	Immunological features	Intervention with follow up duration (months)	Clinical response	Immunological response	Treatment-associated adverse events	Outcome
Zaja 2003 (*n = 3)	pSS 33.3% * EMC 66.6% *	Cutaneous 100% Articular 75% Neuropathy100%	CG 156–1,500 mg/dl IgM 1.2 14 g/L Low C4 2 mg/dl RF 79 526 IU/ml	CS + RTX Follow up 9–31 months	Yes	Yes	Mixed cohort	Relapse 33%; Malignancy developed in 66.6 %
Cruz 2006 (n = 9)	EMC 56% MGUS 11% CTD 33% (SS, MCTD)	Cutaneous 89% Articular 100% Neuropathy 67% Nephropathy 44%	Low CG Low C4	CS + AZA 56% CS + CYC 11% ± RTX/ MTX Follow up 19.3 ± 14 months	CR 44% PR 56%	Not described	Not described	Relapse 22%; Progressive PAH due to underlying MCTD in one patient
Saadoun 2006 (*n = 116)	CTD 34% HM 26% EMC 27%	Cutaneous 56% Articular 28% Neuropathy 8% Nephropathy 32%	CG: CTD/HM 1.2 ± 1.6; EMC 0.9 ± 1.7 Type 2 MC 82% Low C4 68.4% RF 58%	CS ± CYC/AZA, PLEX Mean follow up 49.4 ± 41.6 months	Noted in 25%	Not described	Not described	Relapse 52%; 4-6-fold increased risk B-cell NHL; Death 20%
Matignon 2009 (n = 20)	pSS 45% BCL 5% EMC 50%	Cutaneous 80% Articular 35% Neuropathy 30% Nephropathy 85-100%	MG 85% Low C4 95% Low C3 30% RF+ 85%	CS ± CYC/AZA/fludarabine/ chloraminophene/ anti-CD20/ IFN Follow up 3–264 months	Noted in 65%	Noted in 20%	Not described	Renal relapse 35%; Extrarenal relapse 25%; Death 40%
Terrier 2010 (n = 23)	pSS 39% BCL 35% EMC 35%	Cutaneous 83% Articular 35% Neuropathy 52% Nephropathy 30%	CG 0.77 g/L Type II monoclonal IgMκ 74% Low C4 0.04	RTX ± CS/CYC/MMF/AZA/MTX/PLEX Follow up 6 months	CR 73% PR 13%	CR 50% PR 38% NR 13%	Severe infections 26%; Infusion-related reactions 48%	Relapse 55%; Death 13%
Foessel 2011 (*n = 31)	AID 42% LM 12% EMC 39% Infectious (HepB/ Parvo B19) 6%	Cutaneous 90% Neuropathy 45% Nephropathy 36%	CG EMC 2.8 ± 3.1 g/l AID 2.5 ± 2.9 g/l Low C4 100% RF 80%–100%	CS ± RTX/AZA/ CYC/MMF/MTX/IFN/Lenalidomide Median follow up 67.2 (3–240 months)	Yes	Yes	27.6% in the non-malignant cohort	Relapse during CS taper 100%; Disease flare after RTX 6%
Terrier 2012 & 2013 (n = 242)	EMC 48% CTD 31% HM 21%	Cutaneous 83% Articular 40% Neuropathy 52% Nephropathy 35% GI 5% CNS 2% Pulmonary 2%	CG 0.94 ± 1.61 g/L C4 0.07 ± 0.09 C3 0.86 ± 0.31	CS 100% AKA 38% RTX 35% PLEX 17% AZA/MMF 12% Mean follow up 51 ± 54 months	Yes	Yes	Severe infections (23%) in older age >60 years, renal failure, high dose CS	Death 17%
Terrier 2014 (n = 145)	EMC 38% CTD 36% HM 26%	Cutaneous 87% Articular 42% Neuropathy 55% Nephropathy 35%	CG 0.75 ± 1.14 g/L C4 0.06 ± 0.07 C3 0.84 ± 0.33	CS/CS + CYC/ CS + RTX/PLEX Mean follow up 65 ± 54 months	Noted in 70% (n = 73) Relapse with CR 50% (n = 20)	Noted in 36% (n = 31/86) Relapse with complete IR 4% (1/28)	Not described	Relapse within 12 months 28%; Median time to relapse 9.5 months (3-12) after induction
Bryce 2016 (*n = 6)	LPD 66.6% SS+MALT Lymphoma 33.3% EMC 16% GD 16%	Cutaneous 37-75% Nephropathy 37%	Cryocrit 13 % C4 <3 mg/dl RF 56-580 IU/ml	RTX ± R-CHOP/CLB/ CS/PLEX Median follow up 2.3 years ± 2.4 years	Yes	Yes	None	Death 25%
Zaidan 2016 (n = 230)	pSS 22.5% LPD 28.7% EMC 48.8%	Cutaneous 71.3% Articular 28.7% Neuropathy 42.5% GI 11.3% Pulmonary 5%	Type II MC 84% Low C4 45% Low C3 + C4 18%	CS alone 27.6% RTX + CS 21.1% CYC + CS 36.8% CYC + RTX + CS 10.5% Mean follow up 49.9 ± 45.5 months	Noted in 60%	Noted in 31.3%	RTX + CS severe infections 29.1%, BCL/WM 8.9%, Increased early mortality when used first line	Relapse 42.7% with renal flare 75%; Death 24%
Colantuono 2017 (*n = 2)	EMC 5%	Cutaneous 89% Neuropathy 78% Nephropathy 41%	Not described	RTX ± retreatment	Yes	Yes	Infusion-related reactions and infection	Relapse 100%
Marson 2018 (*n = 42)	Not described	Cutaneous 47.8% Neuropathy 54.7% Nephropathy 27%	Pre-apheresis serum CG > 0.5mg/dL or Cryocrit> 0.5%	AT± RTX/CYC/AZA/IV Ig/IV prostanoids/colchicine	Yes	Not described	None	Unable to segregate
Lesniak 2021 (n = 3)	EMC 67% CTD 33%	Cutaneous 100% Articular 100% Neuropathy 100% GI 100%	CG 3.5 ± 1.7g/L Low IgG 319 ± 73mg/dl	RTX ± CS/MMF	Yes	Yes	Infectious pneumonia 67%	Relapse 100%
Fenoglio 2022 (**#**n = 8)	EMC 54.5%	Cutaneous 40% Neuropathy 80% Nephropathy100%	Mean Cryocrit 2.5% C4 4.9 C3 71 RF 467	RTX Mean follow up 38.4 (6–144 months)	Yes	Yes	RTX infusion-related severe reaction with anti-RTX antibodies and infection 12%	Relapse 37 %; Death due to atherosclerosis 37%
Pouchelon 2022 (n = 26)	AID 51% HM 12% EMC 42%	Cutaneous 81% Articular 48% Neuropathy 60% Nephropathy 39% GI 6%	CG 0.54g/L Cryocrit 7.9% Low C4 88% Low C3 25% RF+ 85%	GC/RTX/AKA/belimumab Mean follow up 26 months (IQR 8–38)	Noted in 20%–86%	Noted in <50%	Severe infections with PLEX, anti-CD20 + Belimumab	Not reported
Roubertou 2022 (*n = 21)	SLE 100%	Cutaneous 15-49% Articular 92-93% Neuropathy 12-15% Nephropathy 38-45%	Type III CG 80% Hypergamma- globulinemia 76% Mean Cryoprecipitate 31.1mg/L (8.6-81.8) Low C4 90% Low C3 81% Decreased CH50 81% RF 0%	CS/RTX/AZA/ MTX/CYC Mean follow up 13.2 (±8.9) years	Noted in 92%	Lack of correlation	Cytopenia and drug-induced hepatitis 23%	Relapse 3%
De Vita 2012 (*n = 4)	HCV-unrelated 7% (93% HCV+ in total sample n=57)	Cutaneous 12% Neuropathy 58% Nephropathy 30%	C4 6.62 ± 8.05 mg/dL RF 528.55 ± 840.12iU/ml	GC + RTX GC + AZA/CYC/PLEX Follow up 24 months	Noted in 25%	Yes	Infections, cardiovascular events & hemorrhagic alveolitis 26%	Relapse within 12 - 18 months in RTX switch group/ RTX; Death 11%
Desbois 2020 (*n = 1)	Infectious (HCV) 86% EMC 14%	Cutaneous 100% Neuropathy 86% GI 29%	Median CG 1.35g/L RF +	CS + RTX ± PLEX	Not described	Not described	Not described	Relapse of flare 8 days (2-16 days); Death 57% after median of 3.3 months
Argyropoulou 2020 (n = 71)	pSS 100% (NHL - MALT 48%)	Cutaneous 90% Articular 71.8% Neuropathy 25% Nephropathy 11%	Type II CG 97% Monoclonal gammopathy in 45.5% Low C4 88.6 RF + 95.7%	CS/Hydroxychloroquine/AZA/MTX/CYC/RTX/PLEX	Not described	Not described	Not described	Lymphoma 33% within 5 years CV onset
Michaud 2015 (n = 29)	CTD 28% SS 17% Systemic sclerosis 7% SLE 3%	Cutaneous 94% Nephropathy 39%	Cryofibrinogen 62% (70 ± 174 mg/L) CG 89 ± 124 Low C4 50% Low C3 28%	CS/RTX/CYC/RTX + CYC/AZA/MTX/PLEX	Not described	Not described	Not described	Cryofibrinogenemia is associated with severe phenotype CV and malignancy
Retamozo 2016 (*n = 21)	pSS 100%	Cutaneous 100% Articular 76.2% Neuropathy 71.4%	Cryocrit 6.58 Low C4 95.2% Low C3 55% RF + 85.7% Monoclonal gammopathy 68.4%	Not included Median follow up 128.4 months	Not described	Not described	Not described	Death 6% at mean follow- up 110.5 months; Developed BCL 9%
Galli 2017 (*n = 160)	EMC 43% CTD 46.8% HM 3%	Cutaneous 62% Articular 77% Neuropathy 45% Nephropathy 32%	Cryocrit 2 %(0.5 - 4%) Median C4 4 (2-12 mg/dL) Median RF 109 (20 – 372)	Not included	Not described	Not described	Not described	Shorter treatment exposure influencing persistence of CG in sera
Boleto 2020 (*n = 266)	SLE 28.9%, pSS 10.7% NHL 5.4%, WM 4 % EMC 23.8%	Cutaneous 54.7% Articular 10.1% Neuropathy 49.6% Nephropathy 21.6%	Monoclonal Ig 5.3g/L Median C4 0.16g/ L RF 20.8 UI/mL	Not included	Not described	Not described	Not described	Not reported

*Cohort isolated from ICV; # Inclusive of type I; AE, adverse event; AID, autoimmune disease; AKA, alkylating agents; ASCT, autologous stem cell transplant; AT, apheresis therapy; AZA, azathioprine; BCL, B-cell lymphoma; CNS, central nervous system; CR, complete response; CS, corticosteroids; CTD, connective tissue disease; CG, cryoglobulins; CH50, complement total; CLB, chlorambucil; CV, cryoglobulinemia vasculitis; CYC, cyclophosphamide; EMC, essential mixed cryoglobulinemia; GC, glucocorticoids; GD, Gaucher’s disease; GI, gastrointestinal; HCV, hepatitis C virus; HM, hematological malignancy; IFN, interferon; Ig, immunoglobulins; IR, immunological response; IV, intravenous; LM, lymphoid malignancy; LPD, lymphoproliferative disorders; MC, mixed cryoglobulinemia; MCTD, mixed connective tissue disease; MG, monoclonal gammopathy; MGUS, monoclonal gammopathy of undetermined significance; MM, multiple myeloma; MMF, mycophenolate mofetil; MTX, methotrexate; NHL, non-Hodgkin's lymphoma; NR, no responders; PAH, pulmonary arterial hypertension; PLEX, plasmapheresis; PNS, peripheral nervous system; PR, partial response; RF, rheumatoid factor; RTX, rituximab; SAE, severe adverse events; SLE, systemic lupus erythematosus; SS, Sjögrens syndrome; pSS, primary Sjögrens syndrome; PR, partial response; WM, Waldenström macroglobulinemia.

Of the 27 studies included in this review, 20 provided insights into the risk factors associated with CV relapse. Four studies investigated relapse in type 1 CV-associated MGUS and HM (25–28), while the remaining twenty studies focused on relapse in mixed CV (10, 29–40). Interestingly, seven studies (14, 16, 20–22, 41) failed to identify any identifiable risk factors for relapse. Below is a comprehensive summary of risk factors for relapse in NICV, including type I cryoglobulinemia.

### Risk factors for relapse in type I CV

#### Patient characteristics

In our review, four studies involving a total population of 206 patients (25–28) were explored. The included studies encompassed patients with a wide age range at the time of diagnosis, spanning from 31 to 91 years (25–28). The restricted availability of data made it difficult to accurately capture the age at relapse. However, it is important to highlight that based on the limited data, females (50%) had a greater probability of CV relapses, especially among those with underlying haematological malignancies, when compared to those with MGUS (28) (**Table 2**).

#### Clinical manifestations

Among the studies that were considered, there were differences in the clinical baseline traits of patients who had relapses (**Table 2**). Skin manifestations were observed in a range of 63% to 100% of cases, where purpura was the most common skin symptom reported in 67% to 69% of cases, followed by necrotic ulcers/gangrene in 27% to 55% of cases, and livedo reticularis in 28% of the population. Peripheral neuropathy was observed in 25% to 47% of patients, while vasomotor symptoms, primarily Raynaud’s phenomenon, were present in 25% to 31% of patients. Articular features, predominantly arthralgia and arthritis, were deduced in 19% to 75% of patients, while renal involvement was observed in 14% and 31%, respectively.

#### Immunological parameters

The median cryoglobulin level was 1.55 g/L in a study by Terrier et al. (26). Furthermore, the mean cryoglobulin level was 2.5 g/L in a study by Néel et al. (27). In a study by Sidana et al. (25), the median cryocrit levels were 10% in HM and 5% in MGUS. Specific cryoglobulin patterns are summarized in **Table 2**. Low C3 and C4 were observed in the study by Terrier et al. (26).

#### Treatment, relapse rate, and time to relapse

All the studies that were included used different treatment regimens (**Table 2**). CS and AKA, RTX and CS/AKA, Rituximab and CS, and PLEX have all been associated with relapses. Due to the variety of the included studies, the follow-up period in each study ranged from 46 to 63 months, and the time to relapse varied. In the included studies, 29% (n = 32) of patients did not respond to first-line therapy, and about 28% (n = 30) of patients relapsed following partial improvement (25–28).

After receiving first-line therapy, Terrier et al. observed a median duration to relapse of 12 months (range: 3–144) for MGUS and 13 months (range: 4–59) for hematological malignancies, whereas Lobbes et al. reported relapses taking place between 4 and 18 months after receiving Rituximab (26, 28). The observed relapse rate varied from 13% to 68%, and this variation was explained using several treatment protocols (26). Terrier et al. found that 68% of patients relapsed after responding to AKA-based regimens, and 40% relapsed after receiving RTX-based therapy. Notably, 13% of patients with underlying hematological malignancies and significant levels of cryoglobulin experienced further CV flares after receiving RTX (26). Additionally, one patient experienced a relapse upon re-treatment with RTX (26). Three studies identified adverse reactions to treatment, such as severe infections (13%–27%) (26, 27) and worsened neuropathy brought on by Rituximab (25%) (28).

#### Factors influencing relapse

Type I CV, occurring in conjunction with an underlying lymphoproliferative disorder, often exhibits a pattern of response followed by relapse, necessitating the use of multiple treatment modalities (27). Frequent relapses were observed in patients with non-IgM type I CV treated with CS, necessitating intense therapy with AKA or RTX (28). RTX-associated CV flare was observed in type I CV (26).

#### Prognosis

Compared to IgM cryoglobulin, type I IgG cryoglobulin was linked with more severe CV features (p = 0.006), with notable cutaneous symptoms and eventual renal involvement (27). Cryoglobulin level and renal involvement, two separate predictor variables, were investigated in relation to symptom burden (25, 27). While Néel et al. agreed that extended follow-up durations permitted early discovery and treatment, favorably affecting prognosis (27), Terrier et al. indicated that underlying hematological malignancies were positively related to lower survival (26). Two studies have consistently shown that advanced age significantly affects prognosis. However, the extent of organ damage varied, with neurological involvement (25) and kidney disease (27) being recognized as significant determinants. According to Lobbes et al., Rituximab can regulate monoclonal proteins, accelerating clinical recovery; however, its efficacy in averting relapse was found to be limited (28).

### Risk factors for relapse in type 2 and type 3 MC

Sixteen studies investigated relapses in NICV (sample size n = 927) (10, 18, 19, 23, 24, 29–40) whereas the remaining seven studies (sample size n = 552) were ambiguous on risk factors for relapse (16, 17, 20–22, 41, 42) consisting of a total sample size of 1,479.

#### Patient characteristics

The mean age at diagnosis ranged between 29 and 66 years, with a female predominance of 70.78%. The mean age for renal disease diagnosis in CV was 60 years (30). There were difficulties separating mean age and gender from the total population in three studies (18, 19, 24), and two studies examined all three CV subtypes (35, 37). The individual study characteristics are summarized in **Table 3**. The etiological associations observed were EMC in 40% (n = 529 cases; total 1,320) (10, 18, 21–24, 29–32, 34–39, 41, 42), autoimmune disease/connective tissue disease in 44% (n = 637; total 1,433) (10, 17, 20, 21, 24, 29–42), hematological malignancies/solid tumors in 19.3% (n = 255; total 1,320), with one case of Gaucher’s disease with hypersplenism (10, 18, 21–24, 29–32, 34–39, 41, 42).

#### Clinical manifestations

The baseline characteristics of the included studies indicated that most studies reported cutaneous features, primarily purpura (10, 16, 17, 20, 21, 24, 29–34, 36–39, 41, 42). A subgroup of studies (18.7%, n = 3) exclusively described nephropathy (24, 30, 35) and neuropathy (19, 23, 39) as cardinal features, while a minority of studies (12%, n = 2) focused on articular features (37, 39). In SLE-associated CV, arthralgia was observed as the leading manifestation, followed by skin features (40) (**Table 3**).

#### Immunological parameters

Common immunological features such as high cryoglobulin levels consisting of monoclonal/polyclonal IgM with RF activity preponderant Kappa light chain or polyclonal IgG (38), cryocrit (>0.5% to 13%) (17, 35, 42), along with low serum C4 levels (7, 19–21, 23, 24, 29–34, 36, 37, 40, 41) were noted.

#### Treatment, relapse rate, and time to relapse

Different treatment regimens were used across the studies, including CS and AKA, RTX with CS/AKA, Rituximab with CS, and PLEX. The follow-up duration in these studies ranged from 3 months to 13.2 years. Relapse rates varied from 22% to 60%, and the time to relapse ranged between 1 and 80 months across eligible studies (10, 18, 24, 29–35, 37–40). Combination regimens such as CS + RTX or CS + CYC showed efficacy in preventing early relapse. RTX + CS was associated with a reduced relapse rate (31–34). Three studies (31, 32, 34) investigated the impact of different treatment regimens on response rates. One study found that the combination of RTX and CS had better efficacy compared to AKA + CS as first- or second-line therapy (32). Another study reported that a high percentage (91%) of relapses occurred within three months in patients treated with CS, while seven patients (42%) showed a partial response to RTX (31). In a different study, relapse rates of approximately 42.7% were observed, with renal relapses being particularly prevalent (75%), in patients treated with CYC + CS (n = 11/20) or CYC + RTX + CS (n = 13/21) (34). A few studies demonstrated that the combination of RTX and CS as a treatment regimen resulted in a decreased relapse rate compared to other treatment approaches (31–34). However, PLEX was found to be ineffective in patients with peripheral neuropathy (16).

De Vita et al. discussed the superiority of RTX to conventional treatment (consisting of CS, AZA, CYC, or PLEX) in refractory cases where subsequent responses were achieved on RTX retreatment. Patients who were switched to the RTX group after showing resistance to non-RTX regimens had an increased risk of clinical relapse. This group, referred to as the “RTX switch group,” consisted of randomly assigned patients who had not responded adequately to previous non-RTX treatments (16). Moreover, Desbois et al. observed that 3.4% of disease flares were specifically linked to Type II mixed cryoglobulinemia. Patients who encountered vasculitis flares following RTX treatment demonstrated a lower 1-year survival rate compared to those who did not experience such flares (20). In three studies, the determination of relapse rates was limited due to the inclusion of cohorts with mixed characteristics, making it difficult to ascertain relapse rates, or due to a combination of the literature review and the original study (19, 23, 36).

#### Factors influencing relapse

Terrier et al. demonstrated that certain factors observed at baseline, including cutaneous necrosis, purpura, and joint involvement, were associated with an elevated risk of experiencing a relapse within a 12-month period. Furthermore, the lack of achieving a complete clinical response (p = 0.034) and immunological response after treatment (p = 0.0005) was also associated with an increased risk of relapse, highlighting the importance of achieving both clinical and immunological responses to reduce the risk of relapse (10). Debois et al. investigated potential factors contributing to RTX-associated vasculitis, where patients who experienced relapses had a higher likelihood of renal involvement (p = 0.0008), B-cell lymphoproliferation (p = 0.015), elevated cryoglobulin levels (2.1 *vs* 0.4 g/l, p = 0.0004), and decreased levels of IgG (2.9 *vs* 10.1 g/l, p = 0.005) and C4 prior to receiving RTX (0.02 *vs* 0.05, p = 0.023), compared to those who did not experience a flare after RTX treatment (22). Foessel et al. observed that there was an increased occurrence of relapses in patients who were treated solely with CS, on tapering, or in those who received a combination of CS and CYC. Moreover, RTX-associated CV flare was also observed (31). This finding is consistent with the study conducted by Terrier et al., indicating that the use of corticosteroids alone (p = 0.026) may be associated with a higher risk of relapse (10).

Several studies have discussed the occurrence of renal (membranous glomerulonephritis) ± CV relapses, particularly in cases where the glomerular filtration rate (GFR) is below 60 ml/min (30, 31, 34). Foessel et al. reported a relapse involving cutaneous manifestations in a patient with underlying EMC (31). Additionally, there have been observations of refractory cases of EMC, especially when treated with non-RTX + CS regimens. Terrier et al. reported that patients with pSS experienced more relapse than patients with EMC (38). pSS-CV patients had a higher frequency of extra glandular manifestations and B-cell lymphoma, which were attributed to underlying Type II IgMκ with RF (20).

#### Prognosis

The mortality rate was higher in pSS (31), and an increased risk of B-cell lymphoproliferative disorder was observed (29). Furthermore, older age as well as renal, pulmonary, and gastrointestinal involvement were found to be poor prognostic factors due to their increased association with severe infections (33). A patient who had MALT and pSS had a relapse of lymphoma, which resulted in subsequent death (24). Another study demonstrated a 4- to 6-fold increased risk of developing B-cell NHL with NICV and low IgG levels (29). Fenoglio et al. reported an increased rate of relapses in type I CV and delineated the development of anti-RTX antibodies in a patient with type III cryoglobulinemia (35).

Inarguably, most relapses are concordant with an incomplete response or refractoriness to treatment, attributed to persistently low C4 levels and high levels of circulating cryoglobulin. Multiple relapses were deduced in patients who were treated with different second-line treatment regimens (33, 34, 39).

#### Studies comparing ICV and NICV

The remaining studies (n = 4) provided comparisons of both ICV and NICV cohorts (21), with minimal emphasis on risk factors for relapse (17, 21, 41, 42). Boleto et al. described the spectrum of both HCV and non-infectious MC in the era of direct antiviral agents (21). Two studies focused on the classification criteria and clinical spectrum of pSS with concomitant CV, respectively (17, 20). Michaud et al. provided unique insights on cryofibrinogenemia as a marker of the severe CV phenotype (41). Galli et al. critically analyzed the influence of clinical and immunological parameters on overall outcomes in NICV, emphasizing poor prognosis in the male gender, the presence of purpura, and/or type II mixed cryoglobulins irrespective of the underlying disease (42). Nonetheless, a large proportion of studies recognized type II MC as a significant factor influencing outcome (17, 20, 22, 42). Retamozo et al. demonstrated a higher mean cryocrit level, a higher frequency of monoclonal gammopathy (68% *vs*. 29%, P = 0.009), and lymphopenia (47.6% *vs*. 20.9%, p = 0.042) among patients with CV (17).

## Discussion

NICV is commonly associated with non-infectious underlying causes, such as autoimmune diseases and hematological malignancies, in contrast to HCV-related CV. Treatments aiming at eliminating HCV infection have reduced relapse risk. In our systematic review, by synthesizing and analyzing existing evidence, we categorized risk factors for relapse in NICV based on Ig isotype. Due to the heterogeneity of the included studies, we delineated risk factors for relapse by identifying common patterns through the correlation of their clinical and immunological responses to various therapeutic approaches across the literature. Notably, each underlying condition impacted the clinical severity and cryoprecipitation in NICV (42).

Our analysis showed that the mean age at diagnosis of NICV was ≥60 years, and the male gender had poor survival outcomes, particularly if there was renal involvement (33, 39, 42). Terrier et al. (2014) described earlier relapses within 12 months in patients with a GFR <60 ml/min/1.73 m^2^ (10).

Type I cryoglobulinemia with an underlying lymphoproliferative disorder tended to be more frequently associated with severe skin manifestations ranging from purpuric lesions to skin necrosis to ulcers in comparison to MC (25–28). Type I cryoglobulinemia lacked RF activity by conventionally abstaining from complement activation (26). In contrast, Kolopp-Sarda et al. suggested possible complement engagement and complement-inducing vasculopathy in type I cryoglobulinemia (43). There is an increased prevalence of hyperviscosity in type I, although some studies have reported leukocytoclastic vasculitis on histopathology, which is typical for mixed CV (44).

A huge proportion of the study population had an underlying autoimmune disease in the spectrum of NICV, where pSS was the leading etiology, followed by SLE. These often present with cutaneous manifestations (17, 20, 21, 32, 40). Additionally, few studies have associated symptoms such as low-grade fever, purpuric lesions, skin ulcers, low C4 complement level, and higher frequency of CV and severity of disease activity in pSS with concomitant cryoglobulin in sera with an increased risk of lymphoma and/or death (17, 20, 45).

Interestingly, Type II MC with monoclonal IgM is more susceptible to precipitation upon cold exposure. This is probably due to the pentameric conformation of IgM exhibiting increased avidity and the presence of multiple Fc sites on the same IgM molecule, making it accessible for rapid and/or multiple C1q binding, subsequently activating the complement pathway. Furthermore, binding affinity properties, glycosylation, amino acid formation, and temperature-induced molecular changes favor cryoprecipitation of IgM (46).

The persistence of cryoglobulinemia despite treatment constitutes a significant risk factor for relapse in NICV. Potential mechanisms for inadequate clearance include defective complement C1q receptor (47), failure of crosstalk between antibodies and complement non-interference with immune precipitation via the classical pathway, and impaired solubilization of immune precipitates impeding clearance by the alternative complement pathway in SLE (48). Circulating cryoglobulins activate complements, forming immune complexes with subsequent complement C4 consumption. Another plausible mechanism postulated for low C4 levels is competent cryoglobulin interaction with the C1q protein (47).

More than 40% of cases were EMC with type III cryoglobulinemia in the GISC study (42), compared to the CryoVas French series (10, 32, 33). Furthermore, most patients with type III EMC progressed to type II during their follow-up, highlighting that ineffective treatment of EMC could impact the clearance of cryoglobulin (42). In our review, frequent relapses were encountered in patients with underlying EMC (23, 31, 37, 39).

A study by Fayed et al. identified predictors of relapses following sustained viral response in HCV-related CV. The main predictors determined the presence of severe clinical manifestations at the end of direct-acting antiviral therapy such as skin ulcers, renal disease, peripheral neuropathy, and male gender (49). These findings align with our review of NICV, which also demonstrated that severe skin manifestations were associated with relapse, while male gender and renal disease were linked to a poorer prognosis. Moreover, the study conducted by Fayed et al. did not find a significant association between relapse and B-cell lymphoma or immunological parameters due to the limited availability of data (49).

The reactivation of RF-expressing B cells occurs when B-cell receptor (BCR) binds to immune complexes and Toll-like receptor-7 (TLR-7) and Toll-like receptor-9 (TLR-9), in response to microbial nucleic acid (50). Unlike TLR7, which had a limited impact on relapses, TLR9 had a significant role in disrupting immune tolerance and promoting relapse, even in patients who had successfully eliminated the virus (51). HCV-related CV patients who initially responded to B-cell targeting agents such as RTX experienced relapses due to ongoing viral mutations, leading to the emergence of different B-cell clones (52). Elevated levels of TNF ligand superfamily member 13B (also known as the B-cell-activating factor of the TNF family [BAFF]) and TNF ligand superfamily member 13 (also known as a proliferation-inducing ligand [APRIL]), which are implicated in B-cell survival and autoimmunity, have been observed in both HCV infection and autoimmune diseases (53). Considering the underlying pathogenic mechanisms, B-cell proliferation is a potential risk factor for relapse. Furthermore, BAFF and APRIL may serve as potential biomarkers to predict disease progression and/or relapse (49).

Several studies explored various therapeutic regimes; conspicuously, the RTX and CS regimen on induction, followed by maintenance RTX, demonstrated better efficacy and safety than CS or AKA, particularly in relapsing and/or refractory NICV patients (10, 23–26, 30–34, 37–40). RTX-associated CV flares have been observed in some patients and may be considered a risk factor for relapse, highlighting the importance of individualized treatment strategies (22, 26, 31). Strikingly, patients who are refractory to RTX share similar characteristics such as female predominance, age >50 years, type II cryoglobulinemia with monoclonal IgM kappa component, pSS, and EMC (32, 36). Saadoun et al. demonstrated significant improvement with the belimumab and RTX combination regimen in refractory cases (54). Further studies are needed to investigate the safety and efficacy of plausible treatment regimens for RTX-refractory cases.

A study by Lobbes et al. observed that patients with type I cryoglobulinemia who received treatment with Rituximab and experienced a reduction in circulating cryoglobulin levels showed rapid clinical improvement (28). Factors including advancing age with renal impairment were associated with poor prognosis (19, 27), and monitoring of cryocrit levels in type I predicted symptom improvement, serving as a prognostic tool in guiding management (25).

Our study has limitations due to the rarity of NICV, cohort size, and heterogeneity of the included studies. Most studies were retrospective observational studies discussing relapses of NICV and the efficacy of multiple treatment regimens. RCTs and/or prospective studies on relapse with a larger sample size are necessary to achieve explicit results. Some studies had mixed cohorts inclusive of ICV, alluding to the possibility of segregation bias. Case reports and case series of less than four were excluded; articles in languages other than English were excluded, indicative of selection and language bias.

To our knowledge, this is the first systematic review exploring identifiable risk factors for relapse with an underlying immunopathological etiology in NICV in relation to Ig isotype. Given the disease’s rarity and lack of RCTs and prospective studies, we could not perform a meta-analysis. Nonetheless, the achievement of remission in HCV-related CV with direct-acting antiviral agents paves the way for NICV as the next leading cause of CV (21). Abating the likelihood of relapse at the time of diagnosis is cumbersome; however, identification of those at risk might be critical for efficiently treating patients with NICV.

## Conclusion

In conclusion, type I NICV accompanied by lymphoproliferative disorder often shows a response-relapse pattern, particularly when treated with CS alone. In MC, baseline factors such as cutaneous necrosis, purpura, and joint involvement, along with incomplete clinical and immunological responses to treatment, increase the risk of relapse. CS monotherapy is associated with a higher relapse risk. RTX-associated CV flare is more common in patients with renal involvement, B-cell lymphoproliferation, elevated cryoglobulin levels, decreased IgG levels, and low C4 prior to RTX treatment. These findings emphasize the importance of achieving both clinical and immunological responses and identifying relapse risk factors in NICV. Further research is needed to optimize treatment strategies and improve long-term outcomes for patients with NICV and associated lymphoproliferative disorders.

## Author contributions

Conceptualization: NR. Data curation: NR and PR. Formal analysis: NR, PR, and HA. Methodology: NR and PR. Validation: NR and PR. Visualization: NR and PR. Writing: original draft: NR and PMR. Writing: review and editing: NR, PR, and HA. All authors contributed to the article and approved the submitted version.
