# Supplementary material for: Risk factors for relapse in non-infectious cryoglobulinemic vasculitis, including type I cryoglobulinemia: a systematic review
Source: Front Immunol. 2023 Jul 7;14:1215345. doi: 10.3389/fimmu.2023.1215345 (PMC10361750; doi:10.3389/fimmu.2023.1215345)
Supplement: Supplementary file 1 [file DataSheet_1.docx]

S1. Sample Data Extraction Form

| **DATA EXTRACTION FORM** | |  |
| --- | --- | --- |
| REVIEWER: |  |  |
| ARTICLE TYPE: |  |  |
| ARTICLE TITLE: |  |  |
| AUTHOR(S)- FIRST AUTHOR: |  |  |
| YEAR OF PUBLICATION: |  |  |
| COUNTRY OF PUBLICATION: |  |  |
| LANGUAGE: |  |  |
| STUDY POPULATION:   - AGE - GENDER - RESEARCH SITE - PREVALENCE/INCIDENCE |  |  |
|  |  |  |
|  |  |  |
|  |  |  |
| STUDY TYPE: |  |  |
| STUDY FINDINGS: |  |  |
| TIMEFRAME: |  |  |
| CLINICAL FEATURES OF CRYOGLOBULINAEMIC VASCULITIS (CV): |  |  |
| DIAGNOSTIC INVESTIGATIONS: |  |  |
| TREATMENT(S): |  |  |
| TREATMENT OUTCOMES:   - COMPLICATIONS - RELAPSE - PROGNOSIS - MORTALITY |  |  |
| OTHER FINDINGS RELEVANT TO THE RESEARCH: |  |  |
| NOTES: |  |  |
