# Supplementary material for: Risk factors for relapse in non-infectious cryoglobulinemic vasculitis, including type I cryoglobulinemia: a systematic review
Source: Front Immunol. 2023 Jul 7;14:1215345. doi: 10.3389/fimmu.2023.1215345 (PMC10361750; doi:10.3389/fimmu.2023.1215345)
Supplement: Supplementary file 2 [file DataSheet_2.docx]

**S2. Newcastle – Ottawa Quality Assessment Scale**

| **Newcastle-Ottawa Quality Assessment Scale for Case-Control studies included in the Systematic Review** | | | | | | | | | |
| --- | --- | --- | --- | --- | --- | --- | --- | --- | --- |
|  | **SELECTION** | | | | **COMPARABILITY** | **EXPOSURE** | | | **Total Score** |
| **Author** | **Is the case definition adequate?** | **Representativeness of the cases** | **Selection of Controls** | **Definitions**  **of Controls** | **Comparability basis of design or analysis** | **Ascertainment of exposure** | **Same**  **method of ascertainment**  **for cases**  **and controls** | **Non-response rate** |  |
| Argyropoulou 2020 | ☆ | ☆ | ☆ | ☆ | ☆ |  |  |  | 5 |
| **Newcastle-Ottawa Quality Assessment Scale for Cohort studies included in the Systematic Review** | | | | | | | | | |
|  | **SELECTION** | | | | **COMPARABILITY** | **OUTCOME** | | | **Total Score** |
| **Author** | **Representativeness of exposed cohort** | **Selection of non-exposed cohort** | **Ascertainment of exposure** | **Outcome of interest not present at start of study** | **Comparability of cohorts based on design or analysis** | **Assessment of outcome** | **Sufficient follow-up time** | **Adequacy of follow up** |  |
| Zaja 2003 |  |  |  | ☆ |  | ☆ | ☆ | ☆ | 4 |
| Bryce 2006 | ☆ |  | ☆ | ☆ |  | ☆ | ☆ |  | 5 |
| Cruz 2006 | ☆ |  | ☆ | ☆ |  | ☆ |  |  | 4 |
| Saadoun 2006 | ☆ |  | ☆ | ☆ |  | ☆ | ☆ |  | 5 |
| Matignon 2009 | ☆ |  | ☆ | ☆ |  | ☆ | ☆ | ☆ | 6 |
| Terrier 2010 | ☆ |  | ☆ | ☆ |  | ☆ | ☆ |  | 5 |
| Foessel 2011 | ☆ |  | ☆ | ☆ |  | ☆ | ☆ |  | 5 |
| Terrier 2012 | ☆ |  | ☆ | ☆ |  | ☆ | ☆ | ☆ | 6 |
| Terrier  2013 (n=64) | ☆ |  | ☆ | ☆ |  | ☆ | ☆ | ☆ | 6 |
| Terrier  2013 (n=242) | ☆ |  | ☆ | ☆ |  | ☆ | ☆ | ☆ | 6 |
| Terrier 2014 | ☆ |  | ☆ | ☆ |  | ☆ | ☆ | ☆ | 6 |
| Néel 2014 | ☆ |  | ☆ | ☆ |  |  | ☆ | ☆ | 5 |
| Michaud 2015 | ☆ | ☆ |  | ☆ | ☆ |  |  |  | 4 |
| Retamozo 2016 | ☆ | ☆ | ☆ | ☆ | ☆ | ☆ |  |  | 6 |
| Zaidan 2016 | ☆ |  | ☆ | ☆ |  | ☆ | ☆ | ☆ | 6 |
| Galli 2017 | ☆ |  |  | ☆ |  |  | ☆ |  | 3 |
| Sidana 2017 | ☆ |  | ☆ | ☆ |  |  | ☆ |  | 4 |
| Lobbes 2018 | ☆ |  | ☆ | ☆ |  |  | ☆ | ☆ | 5 |
| Marson 2018 | ☆ |  | ☆ | ☆ |  | ☆ | ☆ |  | 5 |
| Boleto 2020 | ☆ |  | ☆ | ☆ |  |  |  |  | 3 |
| Desbois 2020 | ☆ | ☆ | ☆ |  | ☆ | ☆ |  |  | 5 |
| Lesniak 2021 | ☆ |  |  |  |  | ☆ | ☆ | ☆ | 4 |
| Fenoglio 2022 | ☆ |  |  | ☆ |  | ☆ | ☆ |  | 4 |
| Pouchelon 2022 | ☆ |  | ☆ | ☆ |  | ☆ | ☆ | ☆ | 6 |
| Roubertou 2022 | ☆ |  | ☆ | ☆ |  |  |  |  | 3 |
