# Supplementary material for: Risk factors for relapse in non-infectious cryoglobulinemic vasculitis, including type I cryoglobulinemia: a systematic review
Source: Front Immunol. 2023 Jul 7;14:1215345. doi: 10.3389/fimmu.2023.1215345 (PMC10361750; doi:10.3389/fimmu.2023.1215345)
Supplement: Supplementary file 3 [file DataSheet_3.docx]

**S3. Quality assessment of RCT using** **Cochrane Collaboration’s Risk of Bias Assessment Tool Version 2 (RoB 2)**

| **Risk of Bias**  **Assessment Tool**  **(Cochrane Library)** | **Randomisation process** | **Deviations from the intended intervention** | **Missing outcome data** | **Measurement of outcome** | **Selection of reported result** | **Overall** |
| --- | --- | --- | --- | --- | --- | --- |
| Colantuono 2017 | 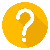 | 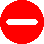 | 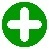 | 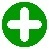 | 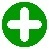 | 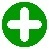 |
| De Vita 2012 | 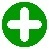 | 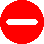 | 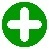 | 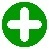 | 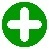 | 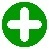 |


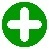
 **Low risk**


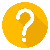
 **Some concerns**


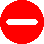
 **High risk**
