# Supplementary material for: Risk factors for relapse in non-infectious cryoglobulinemic vasculitis, including type I cryoglobulinemia: a systematic review
Source: Front Immunol. 2023 Jul 7;14:1215345. doi: 10.3389/fimmu.2023.1215345 (PMC10361750; doi:10.3389/fimmu.2023.1215345)
Supplement: Supplementary file 4 [file DataSheet_4.docx]

S4. STROBE Statement—checklist of items that should be included in reports of observational studies

|  | **Item No.** | **Recommendation** |
| --- | --- | --- |
| **Title and abstract** | 1 | (*a*) Indicate the study’s design with a commonly used term in the title or the abstract  Systematic literature review as stated in the Abstract, Materials and Methods sections page 2, 4,5 |
|  |  | (*b*) Provide in the abstract an informative and balanced summary of what was done and what was found  Provided in the Abstract page 2 |
| **Introduction** |  |  |
| Background/ rationale | 2 | Explain the scientific background and rationale for the investigation being reported  Included in the Background section page 3,4 |
| Objectives | 3 | State specific objectives, including any prespecified hypotheses  Included in the Abstract and Background sections page 2 & 3 |
| **Methods** |  |  |
| Study design | 4 | Present key elements of study design early in the paper  Included in the Methods, Search strategy and Data Extraction sections page 4 - 6 |
| Setting | 5 | Describe the setting, locations, and relevant dates, including periods of recruitment, exposure, follow-up, and data collection  Included in Methods and Search strategy sections page 4 - 6 |
| Participants | 6 | (*a*) *Cohort study*—Give the eligibility criteria, and the sources and methods of selection of participants. Describe methods of follow-up  *Case-control study*—Give the eligibility criteria, and the sources and methods of case ascertainment and control selection. Give the rationale for the choice of cases and controls  *Cross-sectional study*—Give the eligibility criteria, and the sources and methods of selection of participants  Included in Methods and Search strategy sections page 4 - 6 |
|  |  | (*b*) *Cohort study*—For matched studies, give matching criteria and number of exposed and unexposed  *Case-control study*—For matched studies, give matching criteria and the number of controls per case  Not applicable |
| Variables | 7 | Clearly define all outcomes, exposures, predictors, potential confounders, and effect modifiers. Give diagnostic criteria, if applicable  Included in Methods and Search strategy sections page 4 - 6 |
| Data sources/ measurement | 8* | For each variable of interest, give sources of data and details of methods of assessment (measurement). Describe comparability of assessment methods if there is more than one group  Included in Methods and Search strategy sections page 4 - 6 |
| Bias | 9 | Describe any efforts to address potential sources of bias  Addressed in Strengths and Limitations section page 11 |
| Study size | 10 | Explain how the study size was arrived at  Included in Methods and Search strategy sections page 4 - 6 |

| Quantitative variables | 11 | Explain how quantitative variables were handled in the analyses. If applicable, describe which groupings were chosen and why  Included in Methods and Search strategy sections page 4 - 6 |
| --- | --- | --- |
| Statistical methods | 12 | (*a*) Describe all statistical methods, including those used to control for confounding  Included in Methods and Search strategy sections page 4 - 6 |
|  |  | (*b*) Describe any methods used to examine subgroups and interactions  Included in Methods and Search strategy sections page 4 – 6 |
|  |  | (*c*) Explain how missing data were addressed  Not applicable |
|  |  | (*d*) *Cohort study*—If applicable, explain how loss to follow-up was addressed  *Case-control study*—If applicable, explain how matching of cases and controls was addressed  *Cross-sectional study*—If applicable, describe analytical methods taking account of sampling strategy  Included in Methods and Search strategy sections page 4 - 6 |
|  |  | (*e*) Describe any sensitivity analyses  Not applicable |
| **Results** |  |  |
| Participants | 13* | (a) Report numbers of individuals at each stage of study—eg numbers potentially eligible, examined for eligibility, confirmed eligible, included in the study, completing follow-up, and analysed  Included in the Results section page 6 - 10 |
|  |  | (b) Give reasons for non-participation at each stage  Not applicable |
|  |  | (c) Consider use of a flow diagram  Study selection process for systematic review summarised in a flow diagram page 6 |
| Descriptive data | 14* | (a) Give characteristics of study participants (eg demographic, clinical, social) and information on exposures and potential confounders  Included in the Results section page 6 - 11 |
|  |  | (b) Indicate number of participants with missing data for each variable of interest  Not applicable |
|  |  | (c) *Cohort study*—Summarise follow-up time (eg, average and total amount)  Included in the Results section page 6 - 11 |
| Outcome data | 15* | *Cohort study*—Report numbers of outcome events or summary measures over time |
|  |  | *Case-control study—*Report numbers in each exposure category, or summary measures of exposure |
|  |  | *Cross-sectional study—*Report numbers of outcome events or summary measures  Included in the Results section page 6 - 11 |
| Main results | 16 | (*a*) Give unadjusted estimates and, if applicable, confounder-adjusted estimates and their precision (eg, 95% confidence interval). Make clear which confounders were adjusted for and why they were included  Included in the Results section page 6 - 11 |
|  |  | (*b*) Report category boundaries when continuous variables were categorized  Included in the Results section page 6 - 11 |
|  |  | (*c*) If relevant, consider translating estimates of relative risk into absolute risk for a meaningful time period  Not applicable |

| Other analyses | 17 | Report other analyses done—eg analyses of subgroups and interactions, and sensitivity analyses  Not applicable |
| --- | --- | --- |
| **Discussion** |  |  |
| Key results | 18 | Summarise key results with reference to study objectives  Included in the Discussion page 9-11 |
| Limitations | 19 | Discuss limitations of the study, taking into account sources of potential bias or imprecision. Discuss both direction and magnitude of any potential bias  Included in the Discussion page 11 |
| Interpretation | 20 | Give a cautious overall interpretation of results considering objectives, limitations, multiplicity of analyses, results from similar studies, and other relevant evidence  Included in Discussion page 9-11 |
| Generalisability | 21 | Discuss the generalisability (external validity) of the study results  Included in the Discussion page 9-11 |
| **Other information** | |  |
| Funding | 22 | Give the source of funding and the role of the funders for the present study and, if applicable, for the original study on which the present article is based  Provided in text page 12 |
